# Birds Flush Early and Avoid the Rush: An Interspecific Study

**DOI:** 10.1371/journal.pone.0119906

**Published:** 2015-03-23

**Authors:** Diogo S. M. Samia, Daniel T. Blumstein

**Affiliations:** 1 Laboratory of Theoretical Ecology and Synthesis, Department of Ecology, Federal University of Goiás, Goiânia, Goiás, Brazil; 2 Department of Ecology and Evolutionary Biology, University of California Los Angeles, Los Angeles, California, United States of America; University of Western Australia, AUSTRALIA

## Abstract

Since 1986, studies about the escape decisions made by prey are grounded in optimal escape theory (OET) which states that prey will initiate escape when the risk of remaining and the costs of leaving are equal. However, a recent hypothesis, Flush Early and Avoid the Rush (FEAR), acknowledged that the cost of monitoring approaching predators might be a ubiquitous cost. The FEAR hypothesis predicts that prey will generally flee soon after they detect a predator so as to minimize the costs incurred by monitoring the predator. Knowing whether animals flee to reduce monitoring costs is of applied interest because wildlife managers use escape behavior to create set-back zones to reduce human-wildlife conflict. Here we provide the most comprehensive assessment of the FEAR hypothesis using data collected from 178 bird species representing 67 families from two continents. The FEAR hypothesis explains escape behavior in 79% of studied species. Because the FEAR hypothesis is a widespread phenomenon that drives escape behavior in birds, alert distance must be systematically incorporated into the design of set-back zones to protect vulnerable species.

## Introduction

Optimal escape theory (OET) states that prey initiate flight at the distance at which the risk of remaining and the cost of flight are equal [1]. Since this seminal paper, hundreds of studies have generated a rich and diverse set of evidence that has documented various factors that influence flight decisions (reviewed in [2–4]). However, a recent hypothesis acknowledged a ubiquitous cost in the trade-off predicted by OET that apparently explains most of variation in prey’s decision to flee: the cost individuals pay to monitor an approaching predator [5]. The “Flush Early and Avoid the Rush” (FEAR) hypothesis states that animals will flee an approaching predator soon after detection in order to minimize costs incurred by monitoring predator behavior [5]. Ongoing monitoring is expected to increase costs by diverting attention away from beneficial activities, as well as by incurring energetic costs [5–7].

As initially formulated, FEAR is agnostic as to whether flight is initiated immediately upon detection or soon after detection. If flight is initiated at the moment of detection, one might question OET. However, the FEAR hypothesis does not require immediate flight, and thus can be consistent with OET, albeit by highlighting the potential importance of on-going monitoring.

Evidence in support of the FEAR hypothesis has come from studies showing positive relationships between alert distance (AD, the predator-prey distance when prey becomes aware of and begins to monitor the predator) and flight initiation distance (FID, the predator-prey distance when escape begins) [8]. A meta-analysis reviewing studies up to January 2012 found support for FEAR hypothesis in birds and mammals by showing a strong positive correlation between FID and AD in most species [9]. Since then, more studies have identified the same positive relationship in other taxa (e.g. [10,11]).

Despite this apparent support for FEAR, we recently reported that testing the FEAR hypothesis using correlational statistics may be inappropriate much of the time because such statistics might violate assumptions of the statistical test (heteroscedasticity), are particularly sensitive to outliers, and because they do not directly test the key FEAR prediction about fleeing soon after detection [12]. For this reason, we developed a new metric, the phi index (Φ), to correctly test the FEAR prediction. Because conclusions one draws about FEAR are metric-dependent [12], and because initial support for the FEAR hypothesis comes from related datasets [8,9,12], a more comprehensive evaluation using an appropriate metric and a larger data set is warranted.

Developing a fundamental understanding of flight decisions is of applied interest because wildlife managers use escape behavior to create set-back zones to reduce human-wildlife conflict [13]. An implicit assumption underlying setback zone design is that OET drives escape behavior [13–16]. However, if animals tend to flush earlier than predicted by traditional OET, it is possible that current set-back zones are too small, what could result in fitness cost to protected species [17,18].

Here we provide the most comprehensive evaluation of the effect of awareness on species fearfulness to date. Using the phi index (Φ), we tested the effect of alert distance on FID of 178 species of birds studied in USA and Australia, representing 127 genera and 67 families.

## Materials and Methods

### Flight initiation distance

FID data were collected in USA and Australia from 1999 to 2005 using a standard protocol (*sensu* [8,19]). Observers identified birds that were foraging or engaged in ‘relaxed behaviors’, such as roosting or preening. Highly vigilant, obviously alarmed or nesting individuals were not approached, nor were endangered species. FID was measured by walking directly towards the subject at 0.5 m/s. Observers were previously trained to maintain speed constant while minimizing excessive vertical movement across a variety of terrains [20,21]. A marker was dropped at the starting point of the approach. Subsequent flags were dropped when the animal first oriented itself towards the approaching human (AD) and when the animal began to flee (FID). In some rare cases animals fled as soon as they detected the approaching human (i.e., FID = AD), but in most cases, animals oriented towards the human for a period of time before fleeing (i.e., FID < AD). The distances between these markers were afterwards measured to the nearest 0.1 m. Observers attempted to avoid resampling individuals by flushing on birds in different geographical locations and not resampling the same location repeatedly. A modest degree of resampling subjects, however, has been shown to not influence the results of studies like this [21].

### Measuring the effect of awareness on species fearfulness

We used the phi index (Φ) to test the effect of awareness on species fearfulness [12]. Φ is a goodness-of-fit metric that measures how close to AD FID is. Importantly, Φ can be used as an effect size metric which provides the magnitude and direction of the effect of AD on FID [12,22]. Φ is a standardized metric (i.e., it ranges from 0 to 1); Φ-values that deviate from 0.5 (the null expectation; analogous to a Pearson’s *r* = 0 in non-constrained relationships) are a robust indication of a species that flushes later (< 0.5) or earlier (> 0.5). Because AD and FID is an envelope constraint, FID can only assume values equal to or lower than its actual AD (a prey cannot run away from a predator before it has detected it). Thus, the significance of Φ is tested using a null model that respects the constraint FID ≤ AD [12]. Null models were tested with 10,000 iterations. *P*-values ≤ 0.05 were considered significant.

### Statistical methods and phylogenetic non-independence

**Fig 1:**
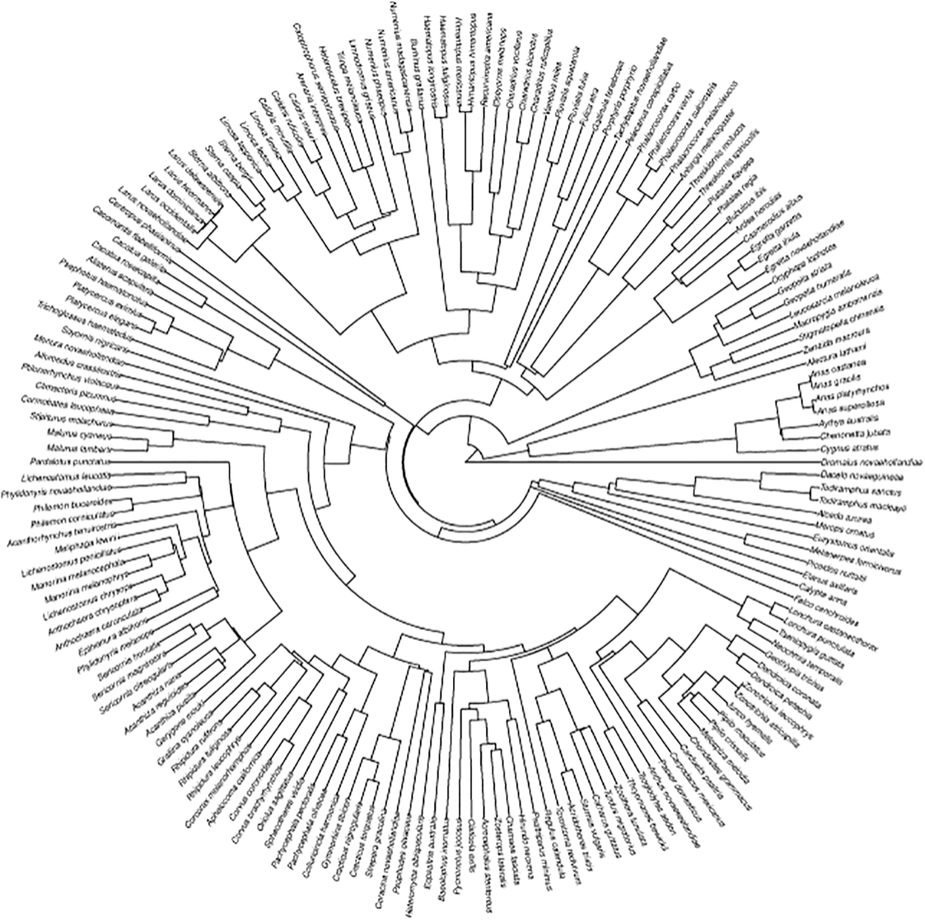
Phylogenetic hypothesis of the 178 avian species included in the present study.

Closely related species are more likely to have similar phenotypes because of their common ancestry. The existence of a phylogenetic structure on animal responses makes observations statistically dependent [23]. We used two metrics to test for phylogenetic signal in the escape response of species from our data set: Blomberg’s K, that assumes Brownian motion character evolution, and a randomization procedure, PIC, which does not assume any underlying model of evolution [24]. Blomberg’s K values range from 0 to infinity; K-values < 1 implies that close relatives resemble each other less than expected under a Brownian motion model [24]. Significance of observed K values was tested with a Monte Carlo test as implemented using the R package “phytools” [25]. The PIC randomization tests if the observed structure in data differs from that expected by chance and was implemented using the R package “picante” [26]. We used the most recent phylogenetic avian hypothesis [27] (Fig. 1).

Of the 178 species studied, 75 were already tested in previous studies [8,9,12]. Therefore, some of our data were not entirely independent so we present results both with (178) and without the 75 species already studied (103).

### Ethics statement

This study was carried out with approval of the Macquarie University Animal Care Committee (protocol # 99021) and the University of California Los Angeles Animal Research Committee (IACUC # 2000–147–01). Data were collected on public and private land after acquiring required permits. By design, experimental approaches were designed to create only a brief disturbance and we are not aware of any lasting harm caused by the experimental approaches. In addition, and to reduce the likelihood of any negative effects, endangered species were not targeted, and we only targeted birds away from their nests.

## Results

### Using the 178 species

**Fig 2:**
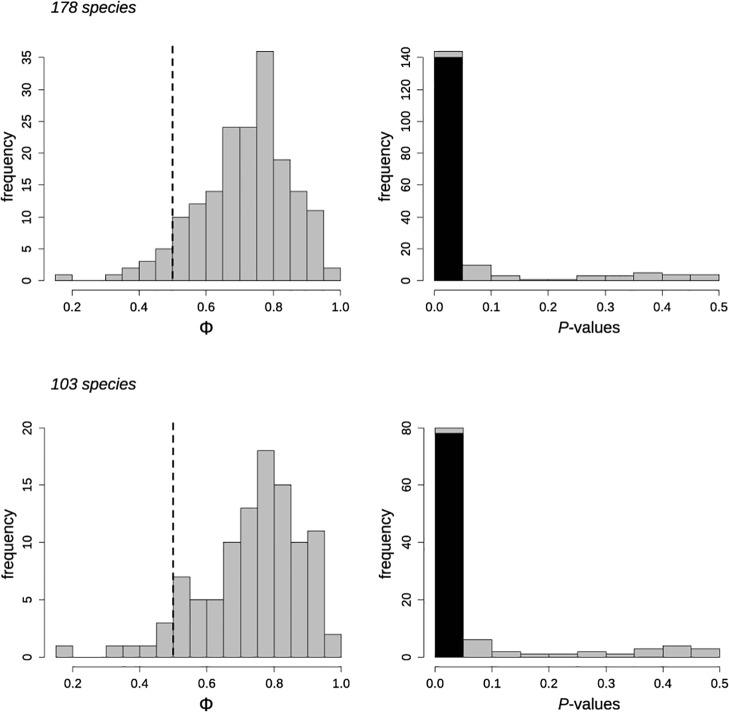
Frequency distribution of phi indices (Φ) and their associated *P*-values of the avian species studied. Results using all species (N = 178) or only the species not tested previously (N = 103) are shown separately. Vertical dashed-line indicates the null expectation of Φ (0.5). Black bars indicate the frequency of species that significantly flushed early (i.e., *P*-values < 0.05).

The Φ-values ranged from 0.16 to 0.97 (Fig. 2; S1 Table) with the most frequent values occurring between 0.75 and 0.8 (36 species; 20%). Overall, 144 of 178 bird species had significant Φ-values: 140 species (79%) significantly flushed early, whereas only four species significantly flushed later. We found no evidence of phylogenetic signal in Φ either using all 178 species (K = 0.06, *P* = 0.32; PIC = 0.003, *P* = 0.29), or using only the 140 species that flushed early (K = 0.03, *P* = 0.49; PIC = 0.004, *P* = 0.50).

### Using only the 103 species not tested in previous study

Overall, the results were quite similar to those using the 178 species. The Φ-values ranged from 0.16 to 0.97 (Fig. 2; S1 Table) with the most frequent values occurring between 0.75 and 0.8 (18 species; 17%). Overall, 80 of 103 bird species had significant Φ-values: 78 species (76%) significantly flushed early, whereas only two species significantly flushed later. Again, there was no evidence of phylogenetic signal in Φ either using all 103 species (K = 0.12, *P* = 0.68; PIC = 0.002, *P* = 0.66), or using only the 78 species that flushed early (K = 0.04, *P* = 0.97; PIC = 0.002, *P* = 0.98).

## Discussion

The Flush Early and Avoid the Rush (FEAR) hypothesis is a recent hypothesis that states that prey flush soon after detecting a predator to reduce monitoring costs. While over 50 years of observations have provided support for Optimal Escape Theory (OET) [1–3], there is less support of the predictions of the FEAR hypothesis. Using a metric specifically designed to quantify how much FID is predicted by AD [12], we showed that most of the 178 bird species tested flushed early (i.e., Φ > 0.5) after detecting an approaching human, in support to FEAR hypothesis. Because many of our Φ-values are less than 1.0, yet greater than 0.5, considerable variation in escape behavior remains to be explained and the rich literature reviewing OET has illustrated how many of the factors that influence the costs and benefits of escape can explain residual variation [2–4].

Importantly, we demonstrated that the overall effect was not biased by results of species previously evaluated [12] since all results were essentially the same with and without the inclusion of the species previously tested. Our present study provides additional and independent results consistent with birds following FEAR’s key prediction.

Future work is needed: 1) to explain why some species may not follow the FEAR hypothesis; 2) to better understand post-escape behavior; 3) to develop formal mathematical models to explain FEAR; and 4) to test the effect of how the predator’s behavior influences FEAR. Comparative analyses should shed light on the first question. Studying post-escape behavior is difficult because in many cases the focal subject flees and is no longer in sight. In some cases, however, animals move away and continue to monitor the approaching human. Understanding the decision to engage in safer on-going monitoring is a question worthy of study because this initially seems to challenge FEAR; why should an animal flush early to reduce on-going monitoring costs only to engage in on-going monitoring? However, FEAR focuses on the decision to flee and on-going monitoring reduces foraging efficiency. It is likely that individuals who maximize foraging efficiency while foraging will have higher expected fitness than those who forage inefficiently; formal models of FEAR can help clarify these tradeoffs. Finally, while this present study approached birds using a standard protocol where the experimenter walked directly towards and looked at the prey, a recent study [28] showed that magpies, *Pica pica*, flushed earlier when looked at directly than when the approacher looked elsewhere. This raises interesting questions: Is this result generalizable to other species? Do prey not directly gazed at flush later due the uncertainty of whether the predator has detected them? Similar unanswered questions apply to predator’s directness of approach.

Interestingly, there was no significant phylogenetic signal in Φ; a finding consistent with previous studies of FEAR [9,12]. The absence of a phylogenetic signal provides insights about the biological factors leading to the flush early phenomenon. If flushing early was overwhelmingly determined by a single factor—such as body size or some other morphological trait—we would expect a strong phylogenetic signal, because such traits are usually highly conserved in most taxa [29,30]. In contrast, the absence of phylogenic signal might suggest that flush early response is influenced by multiple traits. This is because different traits can evolve according different models of evolution (e.g., Brownian Motion, Ornstein-Uhlenbeck, etc.) thereby masking any phylogenetic signal in Φ [31,32]. The lack of a phylogenetic signal may also reflect the importance of context-dependent factors such as predator pressure, current energetic condition, and the degree of habituation to humans—all of which imply that FEAR falls squarely within OET.

Our study confirms that distance at which birds detect a predator is a main determinant in escape decisions made by a diverse set of birds. Other findings support the importance of AD in explaining variation in FID in other taxa. For instance, AD accounted for 72–100% percent of variation of yellow-bellied marmot FID [33]. Likewise, AD was one of the main factors to predict the FID of three large mammals [34].

The general importance of AD in explaining variation in FID has applied implications for those who wish to use escape behavior as the basis of designing set-back zones to reduce human impacts on potentially vulnerable wildlife. Thus, our findings indicate that algorithms used to design set-back zones that seek to minimize human disturbance, should use AD, rather than FID [13,15].

The FEAR hypothesis was originally proposed as a potential general rule in behavioral ecology [5]. Current evidence seems to strongly support FEAR as a widespread phenomenon among birds from different lineages. However, evidence from other taxa were based on a small set of species (e.g., mammals, lizards, snakes and arthropods) (reviewed by [9]). We encourage new research that will permit widespread testing of the FEAR hypothesis in other taxa.

## Supporting Information

S1 Table Summary results of the relationship between alert distance and flight initiation distance of the 178 avian species studied. (PDF)

## References

[1] Ydenberg RC, Dill LM. The economics of fleeing from predators. Adv Study Behav. 1986; 16: 229–247.

[2] Stankowich T, Blumstein DT. Fear in animals: a meta-analysis and review of risk assessment. Proc R Soc B. 2005; 272: 2627–2634. 16321785 10.1098/rspb.2005.3251 PMC1559976

[3] Samia DSM, Blumstein DT, Stankowich T, Cooper WE Jr. Fifty years of chasing lizards: new insights advance optimal escape theory Biol Rev. 2015; In press. 10.1111/brv.12173 25620002

[4] Cooper WE Jr, Blumstein DT. Escaping from predators: an integrative view of escape decisions. 1st ed New York: Cambridge University Press; 2015.

[5] Blumstein DT. Flush early and avoid the rush: a general rule of antipredator behavior? Behav Ecol. 2010; 21: 440–442

[6] Dukas R. Causes and consequences of limited attention. Brain Behav Evol. 2004; 63: 197–210. 15084813 10.1159/000076781

[7] Cooper WE Jr, Blumstein DT. Novel effects of monitoring predators on costs of fleeing and not fleeing explain flushing early in economic escape theory. Behav Ecol. 2014; 25: 44–52.

[8] Blumstein DT. Flight initiation distance in birds is dependent on intruder starting distance. J Wildl Manage. 2003; 67: 852–857.

[9] Samia DSM, Nomura F, Blumstein DT. Do animals generally flush early and avoid the rush? A meta-analysis. Biol Lett. 2013; 9: 20130016 10.1098/rsbl.2013.0016 23426916 PMC3639776

[10] Symonds MRE, Weston MA, Robinson RW, Guay P-J. Comparative analysis of classic brain component sizes in relation to flightiness in birds. PLoS One. 2014; 9: e91960 10.1371/journal.pone.0091960 24637884 PMC3956822

[11] McGowan MM, Patel PD, Stroh JD, Blumstein DT. The effect of human presence and human activity on risk assessment and flight initiation distance in skinks. Ethology. 2014; 120: 1–9. 10.1016/j.pneurobio.2014.05.001 24820404

[12] Samia DSM, Blumstein DT. Phi index: a new metric to test the flush early and avoid the rush hypothesis. PLoS One. 2014; 9: e113134 10.1371/journal.pone.0113134 25405872 PMC4236129

[13] Blumstein DT, Fernández-Juricic E. A Primer of Conservation Behavior. 1st ed Sunderland: Sinauer Associates; 2010.

[14] Rodgers JA, Schwikert ST. Buffer-zone distances to protect foraging and loafing waterbirds from disturbance by personal watercraft and outboard-powered boats. Conserv Biol. 2002; 16: 216–224. 10.1046/j.1523-1739.2002.00316.x 35701971

[15] Fernández-Juricic E, Venier MP, Renison D, Blumstein DT. Sensitivity of wildlife to spatial patterns of recreationist behavior: a critical assessment of minimum approaching distances and buffer areas for grassland birds. Biol Conserv. 2005; 125: 225–235.

[16] Weston MA, Mcleod EM, Blumstein DT, Guay P-J. A review of flight-initiation distances and their application to managing disturbance to Australian birds. Emu. 2012; 112: 269–286.

[17] Preisser EL, Bolnick DI, Bernard MF. Scared to death? The effects of intimidation and consumption in predator-prey interactions. Ecology. 2005; 86: 501–509.

[18] McCoy MW, Bolker BM. Trait-mediated interactions: influence of prey size, density and experience. J Anim Ecol. 2008; 77: 478–486. 10.1111/j.1365-2656.2008.01372.x 18312336

[19] Blumstein DT. Developing an evolutionary ecology of fear: how life history and natural history traits affect disturbance tolerance in birds. Anim Behav. 2006; 71: 389–399.

[20] Blumstein DT, Runyan A, Seymour M, Nicodemus A, Ozgul A, Ransler F, et al Locomotor ability and wariness in yellow-bellied marmots. Ethology. 2004; 110: 615–634.

[21] Runyan AM, Blumstein DT. Do individual differences influence flight initiation distance? J Wildl Manage. 2004; 68: 1124–1129.

[22] Koricheva J, Gurevitch J, Mengersen K. Handbook of meta-analysis in ecology and evolution. 1st ed Princeton: Princeton University Press; 2013.

[23] Felsenstein J. Inferring Phylogenies. 1st ed Sunderland: Sinauer Associates; 2004.

[24] Blomberg SP, Garland T, Ives AR. Testing for phylogenetic signal in comparative data: behavioral traits are more labile. Evolution. 2003; 57: 717–745. 12778543 10.1111/j.0014-3820.2003.tb00285.x

[25] Revell LJ. phytools: an R package for phylogenetic comparative biology (and other things). Methods Ecol Evol. 2012; 3: 217–223.

[26] Kembel SW, Cowan PD, Helmus MR, Cornwell WK, Morlon H, Ackerly DD, et al Picante: R tools for integrating phylogenies and ecology. Bioinformatics. 2010; 26: 1463–1464. 10.1093/bioinformatics/btq166 20395285

[27] Jetz W, Thomas GH, Joy JB, Hartmann K, Mooers AO. The global diversity of birds in space and time. Nature. 2012; 491: 444–448. 10.1038/nature11631 23123857

[28] Lee S-i, Hwang S, Joe Y-e, Cha H-k, Joo G-h, Lee H-j, et al Direct look from a predator shortens the risk-assessment time by prey. PLoS One. 2013; 8: e64977 10.1371/journal.pone.0064977 23755164 PMC3673954

[29] Freckleton RP, Harvey PH, Pagel M. Phylogenetic analysis and comparative data: a test and review of evidence. Am Nat. 2002; 160: 712–726. 10.1086/343873 18707460

[30] Peters RH. The ecological implications of body size. 2nd ed Cambridge: Cambridge University Press; 1986.

[31] Butler MA, King AA. Phylogenetic comparative analysis: a modeling approach for adaptive evolution. Am Nat. 2004; 164: 683–695. 29641928 10.1086/426002

[32] Hansen TF. Stabilizing selection and the comparative analysis of adaptation. Evolution. 1997; 51: 1341–1351. 28568616 10.1111/j.1558-5646.1997.tb01457.x

[33] Williams DM, Samia DSM, Cooper WE Jr, Blumstein DT. The flush early and avoid the rush hypothesis holds after accounting for spontaneous behavior. Behav Ecol. 2014; 25: 1136–1147.

[34] Taylor AR, Knight RL. Wildlife responses to recreation and associated visitor perceptions. Ecol Appl. 2003; 13: 951–963.

