# Supplementary material for: Birds Flush Early and Avoid the Rush: An Interspecific Study
Source: PLoS One. 2015 Mar 23;10(3):e0119906. doi: 10.1371/journal.pone.0119906 (PMC4370843; doi:10.1371/journal.pone.0119906)
Supplement: S1 Table — (PDF) [file pone.0119906.s001.pdf]

Supporting Information

Birds flush early and avoid the rush: an interspecific study

Diogo S. M. Samia[1*], Daniel T. Blumstein[2]

[1]*Laboratory of Theoretical Ecology and Synthesis, Department of Ecology, Federal University of Goiás, Goiânia, Goiás, Brazil*

[2]*Department of Ecology and Evolutionary Biology, University of California Los Angeles, Los Angeles, California,* United States of America

*Corresponding author

E-mail: diogosamia@gmail.com (DSMS)

S1 Table. Summary results of the relationship between alert distance and flight initiation distance of the 178 avian species studied. N, sample size; Φ, the phi index values; and *P*; associated *P*-value of Φ.

**Species**	**Family**	**N**	**Φ**	***P***
Acanthiza nana	Pardalotidae	12	0.74	0.002
Acanthiza pusilla	Pardalotidae	26	0.76	<0.001
Acanthiza reguloides	Pardalotidae	14	0.79	<0.001
Acanthorhynchus tenuirostris $^{-}$	Meliphagidae	36	0.86	<0.001
Acridotheres tristis $^{-}$	Sturnidae	37	0.59	0.029
Acrocephalus stentoreus	Sylviidae	14	0.81	<0.001
Ailuroedus crassirostris	Ptilonorhynchidae	11	0.74	0.001
Alcedo azurea	Alcedinidae	6	0.48	0.410
Alectura lathami	Megapodiidae	11	0.64	0.060
Alisterus scapularis	Psittacidae	7	0.74	0.014
Anas castanea	Anatidae	55	0.85	<0.001
Anas gracilis	Anatidae	22	0.77	<0.001
Anas platyrhynchos	Anatidae	5	0.91	<0.001
Anas superciliosa	Anatidae	50	0.79	<0.001
Anhinga melanogaster	Anhingidae	8	0.49	0.458
Anthochaera carunculata	Meliphagidae	12	0.61	0.088
Anthochaera chrysoptera $^{-}$	Meliphagidae	38	0.67	<0.001
Anthus novaeseelandiae $^{-}$	Motacillidae	55	0.7	<0.001
Aphelocoma californica	Corvidae	25	0.88	<0.001
Ardea herodias	Ardeidae	46	0.65	<0.001
Arenaria interpres $^{-}$	Scolopacidae	42	0.64	<0.001
Aythya australis	Anatidae	5	0.92	<0.001
Baeolophus inornatus	Paridae	9	0.91	<0.001
Bubulcus ibis	Ardeidae	9	0.83	<0.001
Burhinus grallarius	Burhinidae	13	0.79	<0.001
Cacatua galerita $^{-}$	Cacatuidae	34	0.62	0.007
Cacatua roseicapilla $^{+}$	Cacatuidae	120	0.38	<0.001
Cacomantis flabelliformis	Cuculidae	19	0.77	<0.001
Calidris mauri $^{+}$	Scolopacidae	22	0.74	<0.001
Calidris minutilla	Scolopacidae	33	0.69	<0.001
Calidris ruficollis $^{-}$	Scolopacidae	60	0.72	<0.001
Calypte anna	Trochilidae	7	0.75	0.011
Carduelis psaltria	Fringillidae	4	0.89	0.002
Carpodacus mexicanus	Fringillidae	7	0.84	<0.001
Casmerodius albus $^{+}$	Ardeidae	69	0.66	<0.001
Catharus guttatus	Turdidae	6	0.81	0.002
Catoptrophorus semipalmatus	Scolopacidae	92	0.63	<0.001

Centropus phasianinus	Centropodidae	4	0.71	0.071
Chamaea fasciata	Timaliidae	10	0.75	0.003
Charadrius bicinctus	Charadriidae	9	0.66	0.041
Charadrius ruficapillus	Charadriidae	6	0.79	0.005
Charadrius vociferus	Charadriidae	5	0.85	0.002
Chenonetta jubata +	Anatidae	77	0.62	<0.001
Chondestes grammacus	Emberizidae	6	0.7	0.04
Cisticola exilis -	Sylviidae	34	0.83	<0.001
Climacteris picumnus	Climacteridae	12	0.92	<0.001
Colluricincla harmonica	Pachycephalidae	11	0.81	<0.001
Coracina novaehollandiae -	Campephagidae	15	0.85	<0.001
Corcorax melanorhamphos	Corcoracidae	12	0.75	0.002
Cormobates leucophaea	Climacteridae	12	0.86	<0.001
Corvus brachyrhynchos	Corvidae	4	0.77	0.032
Corvus coronoides -	Corvidae	60	0.69	<0.001
Cracticus nigrogularis	Artamidae	5	0.71	0.052
Cracticus torquatus	Artamidae	5	0.71	0.048
Cygnus atratus	Anatidae	18	0.71	<0.001
Dacelo novaeguineae -	Halcyonidae	51	0.56	0.078
Dendroica coronata	Parulidae	25	0.86	<0.001
Dendroica petechia	Parulidae	7	0.52	0.437
Dromaius novaehollandiae	Casuariidae	6	0.93	<0.001
Egretta garzetta	Ardeidae	6	0.8	0.003
Egretta novaehollandiae	Ardeidae	33	0.62	0.009
Egretta thula +	Ardeidae	47	0.57	0.048
Elanus axillaris	Accipitridae	5	0.45	0.343
Elseyornis melanops -	Charadriidae	30	0.75	<0.001
Eopsaltria australis -	Petroicidae	77	0.78	<0.001
Epthianura albifrons	Meliphagidae	11	0.94	<0.001
Eurystomus orientalis	Coraciidae	23	0.69	<0.001
Falco cenchroides	Falconidae	8	0.56	0.297
Fulica atra	Rallidae	8	0.85	<0.001
Gallinula tenebrosa -	Rallidae	33	0.73	<0.001
Geopelia humeralis	Columbidae	3	0.35	0.188
Geopelia striata	Columbidae	8	0.68	0.034
Geothlypis trichas	Parulidae	6	0.9	<0.001
Gerygone mouki -	Pardalotidae	29	0.79	<0.001
Grallina cyanoleuca -	Monarchidae	89	0.7	<0.001
Gymnorhina tibicen +	Cracticidae	115	0.56	0.015
Haematopus fuliginosus	Haematopodidae	59	0.67	<0.001
Haematopus longirostris	Haematopodidae	22	0.61	0.036
Heteromyias albispecularis	Pteroicidae	6	0.56	0.320
Heteroscelus brevipes	Scolopacidae	46	0.66	<0.001
Himantopus himantopus	Recurvirostridae	62	0.76	<0.001

Himantopus mexicanus $^{+}$	Recurvirostridae	52	0.66	<0.001
Hirundo neoxena $^{-}$	Hirundinidae	27	0.69	<0.001
Junco hyemalis	Emberizidae	17	0.81	<0.001
Larus delawarensis $^{+}$	Laridae	19	0.58	0.122
Larus dominicanus	Laridae	12	0.62	0.079
Larus heermanni	Laridae	6	0.55	0.351
Larus novaehollandiae	Laridae	133	0.5	0.504
Larus occidentalis	Laridae	74	0.56	0.033
Leucosarcia melanoleuca	Columbidae	12	0.9	<0.001
Lichenostomus chrysops $^{-}$	Meliphagidae	26	0.75	<0.001
Lichenostomus leucotis	Meliphagidae	5	0.78	0.013
Lichenostomus penicillatus	Meliphagidae	22	0.84	<0.001
Limnodromus griseus	Scolopacidae	11	0.64	0.061
Limosa fedoa	Scolopacidae	35	0.61	0.01
Limosa lapponica	Scolopacidae	92	0.65	<0.001
Limosa limosa	Scolopacidae	6	0.83	0.002
Lonchura castaneothorax	Passeridae	10	0.94	<0.001
Lonchura punctulata $^{-}$	Passeridae	37	0.88	<0.001
Macropygia amboinensis	Columbidae	8	0.47	0.394
Malurus cyaneus $^{-}$	Maluridae	86	0.74	<0.001
Malurus lamberti $^{-}$	Maluridae	36	0.77	<0.001
Manorina melanocephala	Meliphagidae	36	0.42	0.047
Manorina melanophrys	Meliphagidae	44	0.74	<0.001
Melanerpes formicivorus	Picidae	7	0.7	0.032
Meliphaga lewinii $^{-}$	Meliphagidae	27	0.78	<0.001
Melospiza melodia	Emberizidae	17	0.77	<0.001
Menura novaehollandiae	Menuridae	22	0.76	<0.001
Merops ornatus	Meropidae	4	0.16	0.007
Neochmia temporalis $^{-}$	Estrildidae	49	0.82	<0.001
Numenius americanus	Scolopacidae	18	0.71	0.002
Numenius madagascariensis	Scolopacidae	42	0.76	<0.001
Numenius phaeopus	Scolopacidae	27	0.59	0.049
Ocyphaps lophotes $^{-}$	Columbidae	26	0.62	0.015
Oriolus sagittatus $^{-}$	Oriolidae	30	0.76	<0.001
Pachycephala olivacea	Pachycephalidae	6	0.89	<0.001
Pachycephala pectoralis	Pachycephalidae	13	0.81	<0.001
Pardalotus punctatus	Pardalotidae	6	0.53	0.411
Passer domesticus	Fringillidae	11	0.76	0.002
Pelecanus conspicillatus	Pelecanidae	38	0.53	0.296
Phalacrocorax carbo $^{-}$	Phalacrocoracidae	28	0.63	0.008
Phalacrocorax melanoleucos	Phalacrocoracidae	58	0.45	0.090
Phalacrocorax sulcirostris $^{-}$	Phalacrocoracidae	29	0.48	0.385
Phalacrocorax varius $^{-}$	Phalacrocoracidae	23	0.52	0.337

Philemon buceroides	Meliphagidae	7	0.31	0.045
Philemon corniculatus	Meliphagidae	52	0.68	<0.001
Phylidonyris melanops	Meliphagidae	10	0.77	<0.001
Phylidonyris novaehollandiae ⁻	Meliphagidae	45	0.8	<0.001
Picoides nuttallii	Picidae	9	0.85	<0.001
Pipilo crissalis	Emberizidae	44	0.68	<0.001
Pipilo maculatus	Emberizidae	32	0.88	<0.001
Platalea flavipes	Threskiornithidae	4	0.68	0.117
Platalea regia	Threskiornithidae	18	0.66	0.01
Platycercus elegans $^{+}$	Psittacidae	66	0.55	0.074
Platycercus eximius $^{-}$	Psittacidae	24	0.7	<0.001
Pluvialis fulva	Charadriidae	15	0.75	<0.001
Pluvialis squatarola $^{+}$	Charadriidae	42	0.74	<0.001
Porphyrio porphyrio	Rallidae	68	0.76	<0.001
Psaltriparus minimus	Aegithalidae	26	0.92	<0.001
Psephotus haematonotus	Psittacidae	4	0.66	0.134
Psophodes olivaceus $^{-}$	Cinclosomatidae	47	0.81	<0.001
Ptilonorhynchus violaceus $^{-}$	Ptilonorhynchidae	17	0.8	<0.001
Pycnonotus jocosus	Pycnonotidae	17	0.88	<0.001
Recurvirostra americana	Recurvirostridae	5	0.51	0.465
Regulus calendula	Regulidae	10	0.91	<0.001
Rhipidura fuliginosa $^{-}$	Dicruridae	29	0.74	<0.001
Rhipidura leucophrys $^{-}$	Dicruridae	43	0.75	<0.001
Rhipidura rufifrons	Dicruridae	6	0.94	<0.001
Sayornis nigricans	Tyrannidae	6	0.79	0.005
Sericornis citreogularis $^{-}$	Pardalotidae	47	0.79	<0.001
Sericornis frontalis	Pardalotidae	40	0.76	<0.001
Sericornis magnirostra	Acanthizidae	15	0.51	0.462
Sphecotheres viridis	Oriolidae	4	0.7	0.086
Sterna albifrons	Laridae	6	0.58	0.259
Sterna bergii	Laridae	36	0.48	0.371
Sterna caspia	Laridae	11	0.82	<0.001
Stigmatopelia chinensis	Columbidae	52	0.66	<0.001
Stipiturus malachurus	Maluridae	5	0.77	0.015
Strepera graculina $^{-}$	Artamidae	19	0.73	<0.001
Sturnus vulgaris $^{-}$	Sturnidae	27	0.77	<0.001
Tachybaptus novaehollandiae	Podicipedidae	15	0.96	<0.001
Taeniopygia guttata	Estrildidae	6	0.53	0.4
Threskiornis molucca	Threskiornithidae	48	0.66	<0.001
Threskiornis spinicollis	Threskiornithidae	10	0.78	<0.001
Thryomanes bewickii	Troglodytidae	5	0.95	<0.001
Todiramphus macleayii	Alcedinidae	6	0.76	0.015
Todiramphus sanctus	Halcyonidae	10	0.84	<0.001
Toxostoma redivivum	Mimidae	7	0.74	0.014

Trichoglossus haematodus	Psittacidae	7	0.53	0.379
Tringa melanoleuca +	Scolopacidae	7	0.83	<0.001
Troglodytes aedon	Troglodytidae	4	0.84	0.007
Turdus migratorius	Turdidae	13	0.55	0.245
Vanellus miles	Charadriidae	37	0.65	<0.001
Zenaida macroura	Columbidae	12	0.78	<0.001
Zonotrichia atricapilla	Emberizidae	8	0.97	<0.001
Zonotrichia leucophrys	Emberizidae	42	0.69	<0.001
Zoothera lunulata -	Turdidae	24	0.79	<0.001
Zosterops lateralis -	Zosteropidae	29	0.75	<0.001

Note: As mentioned in the main text, data from 75 of 178 species tested in present study were already used in previous studies (compiled in [1]). Some of these species had their sample size increased because of the addition of observations since publication of the original study (11 species; indicated by +), whereas other had their sample size slightly reduced (39 species; indicated by -). This reduction in sample size is justifiable because, after a very careful scrutiny of the data set, we found few questionable observations in some species: some observations were recorded as having a predator's starting distance shorter than prey's alert distance. Such records are very likely typos (because of the methodology used to study escape behavior [2], an animal cannot be alert to an approaching predator before the predator initiates the approach towards a prey).

Specifically, we eliminated 1 observation for each of 10 species; 2 observations for 8 species; 3 observations for 6 species, 4 observations for 5 species; 5 observations for 5 species; 6 observations for 2 species; 7 observations for 2 species; and 11 observations for 1 species. These changes led to either no difference or very small differences of Φ values in both directions (median = 0.01, ranging from 0 [11 species] to 0.05 [1 species]). Importantly, only 3 of 39 species with reduced sample sizes led to changes in the conclusion with respect to their escape strategy; two were no longer significant and 1 became significant.

Supporting Information References

1. Samia DSM, Blumstein DT. Phi index: a new metric to test the flush early and avoid the rush hypothesis. PLoS One. 2014; 9: e113134.

2. Blumstein DT, Samia DSM, Stankowich T, Cooper WE Jr. Best practice for the study of escape behavior. In: Cooper WE Jr, Blumstein DT, editors. Escaping from predators: an integrative view of escape decisions. New York: Cambridge University Press; 2015. pp. 743–768.
